# Association between Dental Caries, Obesity and Salivary Alpha Amylase in Adolescent Girls of Babol City, Iran-2017

**DOI:** 10.30476/DENTJODS.2020.84190.1070

**Published:** 2021-03

**Authors:** Marie Kor, Mahdi Pouramir, Soraya Khafri, Shima Ebadollahi, Samane Gharekhani

**Affiliations:** 1 Student Research Committee, Babol University of Medical Sciences, Babol, Iran; 2 Cellular and Molecular Biology Research Center, Health Research Institute, Babol University of Medical Sciences, Iran; 3 Dept. of Biostatics and Epidemiology, Faculty of Medicine, Babol University of Medical Sciences, Babol, Iran; 4 Student of Clinical Biochemistry, Babol University of Medical Sciences, Babol, Iran; 5 Oral Health Research Research Center, Institute of Health, Babol University of Medical Sciences, Babol, Iran

**Keywords:** Adolescent, Alpha amylase, BMI, DMFT, Saliva

## Abstract

**Statement of the Problem:**

Adolescents are at risk of obesity and caries due to various factors such as diet and poor health habits; these factors may affect their body mass index (BMI) and salivary components. Therefore, it is necessary to assess these factors and their relationship in this age group.

**Purpose:**

This study aimed to evaluate the association between decayed missing filled teeth index (DMFT), salivary alpha amylase (sAA) level and age-specific BMI in adolescent girls.

**Materials and Method:**

A cross-sectional study was conducted on 81 females aged 13-15 years in 3 groups of BMI percentiles; “normal”, “at risk for overweight” and “overweight” (n=27). DMFT was calculated and unstimulated saliva samples were collected. The sAA level was measured with a spectrophotometer. Data were analyzed using Kolmogorov-Smir-nov test, Kruskal- Wallis and Spearman correlation tests using SPSS (version 23) at *p*< 0.05.

**Results:**

The concentration of sAA and mean DMFT were estimated 1326.56±4.73 U/L and 2.77±2.36, respectively. There was no significant difference in sAA level and mean DMFT among BMI groups. A positive and significant correlation was found between sAA and DMFT in overweight group (r 0.46, *p*= 0.014).

**Conclusion:**

Within the limitation of this study, higher levels of sAA may be considered as an indicator for dental caries in overweight adolescent girls.

## Introduction

Adolescence is defined as a critical developmental period of life experienced from the ages of 10 to 24 years [ 1 ]. Unhealthy dietary habits including consumption of industrial foods high in fat, sodium, carbohydrate, and low fiber intake along with low physical activity can affect general heath of adolescents [ 2 ]. Also, the puberty brings stress, emotional imbalance and rebellion for teenagers that influence their lifestyle [ 3 ]. Paying attention to body health of adolescents is a momentous responsibility to prevent serious chronic diseases that may establish later in their life [ 4 ].

The two most prevalent problems among teenagers are dental caries and obesity [ 4 - 5 ]. According to a World Health Organization (WHO) report on 2018 [ 5 ], the worldwide prevalence of obesity for children and adolescents has risen more than ten folds in the last 4 decades. The result of a systematic review conducted by Rahmani *et al*. [ 6 ] showed the prevalence rate of 6.5% for obesity in Iranian children younger than 18. The BMI is calculated using equation [ 7 ]. According to the Center for Disease Control and Prevention and considering the growth fluctuation and gender differences, the BMI for children younger than 20, is plotted against age and sex-specific percentiles. The subjects are defined as” at risk for overweight” If their BMI is between 85th and 95th percentiles, and they are known as clinically overweight if the BMI exceeds the 95th percentile [ 8 ]. Unfortunately, there is a lack of systematic data on the prevalence of dental caries in Iranian adolescents. Pakpour *et al*. [ 9 ] and Yazdani *et al*. [ 10 ] in two separate cross-sectional studies reported DMFT of 2.6 and 2.1, respectively for Iranian teenagers which is higher than the standard (DMFT=1) determined by the WHO [ 11 ].

Obesity and dental caries are both multifactorial phenomena which are influenced by both genetic and environmental factors [ 6 , 12 ]. Despite the correlation established between obesity and tooth decay in adults, the evidence confirming this association in adolescents is controversial [ 13 - 15 ]. It is generally agreed that an etiologic factor common to both tooth decay and obesity is high carbohydrate intake especially in adolescent years [ 14 ]. One of the salivary components related to dental caries is salivary alpha amylase (sAA) [ 16 ]. This enzyme consists at least 50% of salivary proteins and plays a role in starch hydrolysis [ 17 ]. It is demonstrated that this enzyme facilitates the microbial adhesion to enamel surface and acid production [ 16 ]. The level and activity of sAA can be changed under specific situations including stress, anxiety, exercise and circadian rhyth-ms. The higher basal sAA has been found in adolescents with more advanced pubertal development [ 18 ]. However, the association between sAA activity and BMI is controversial [ 19 - 21 ]. Al dossari *et al*. [ 17 ] showed a reverse correlation between sAA and BMI in overwei-ght and obese adults. In contrast, Mennella *et al*. [ 19 ] indicated a higher sAA activity in overweight individuals. Because of the known importance of alpha amylase and the controversies found in various article and also, due to the lack of an article exclusively on the effect of this enzyme during adolescence, the present study aime-d to assess the association between DMFT, sAA and BMI in a selected population of Iranian adolescent girls.

## Materials and Method

This cross-sectional study was carried out on female students in the age range of 13 to 15 years. All cases were randomly selected among the grade 1 of high schools of Babol (north of Iran). Exclusion criteria were adopted as suffering from systemic diseases, consumption of medication over the past month, poor oral hygiene, unwillingness to participate in the study and having orthodontic appliances. The sample size was calculated with consideration of the effect of 4% at the level of x= 0.05 with the power of 80%. Using the software G power, 81 students was calculated (in 3 groups of 27 each one). After approving the study protocol by the Ethic Committee of Babol University of Medical Sciences (MUBABOL. REC.1396.12) and obtaining permission from the Babol Education Department, five high schools were selected randomly for sampling. The height and the weight of students were measured without shoes by a scale (LETO+Italy) and a meter. BMI of students were calculated based on [weight/ (height)^2^] equation and pointed on the age-sex specific BMI percentile chart. Based on the age-sex specific BMI percentile [ 18 ], the selected participants were grouped in 85> BMI>5 (normal weight) 85> BMI> 95(at risk for overweight), and BMI>95(over weight). Oral examination was done by a trained dental student using WHO criteria for caries detection and then the DMFT was recorded [ 22 ]. The teeth that were extracted or repaired due to trauma or extracted because of orthodontic treatment were not included in the DMFT calculation. The unstimulated saliva samples were collected at the morning, at least 2 hours after eating. The students were kept seated and relaxed to allow the saliva to be accumulated in the mouth for 5 minutes. Then, the saliva samples were poured in the containers and were dispatched to the laboratory in the flasks including ice cubes. The amount of sAA was measured using the special kit (Zist Shimi Co., Iran) and spectrophotometric method (Unico, USA). Data were analyzed using Kolmogorov-Smirnov test, Kruskal Wallis test and Spearman correlation coefficient using SPSS (version 23) and *p*< 0.05 was considered statistically significant. The study variables are listed in Table 1.

**Table 1:** Study variables

Variable	Type	Role	Scale
BMI	Nominal qualitative	Independent	CDC’s percentiles
DMFT	Nominal qualitative	Dependent	<3.1
			3.1-4.5
			4.6-6.9
			>6.9
	Ratio quantitative		digit
sAA	Ratio quantitative	Dependent	U/L
Age	Ratio quantitative	Contextual	year

BMI: Body mass index, DMFT (D, M, F, T): Decayed missing filled teeth; CDC: Centers for disease control and prevention; sAA: Salivary alpha amylase

## Results

**Figure 1:**
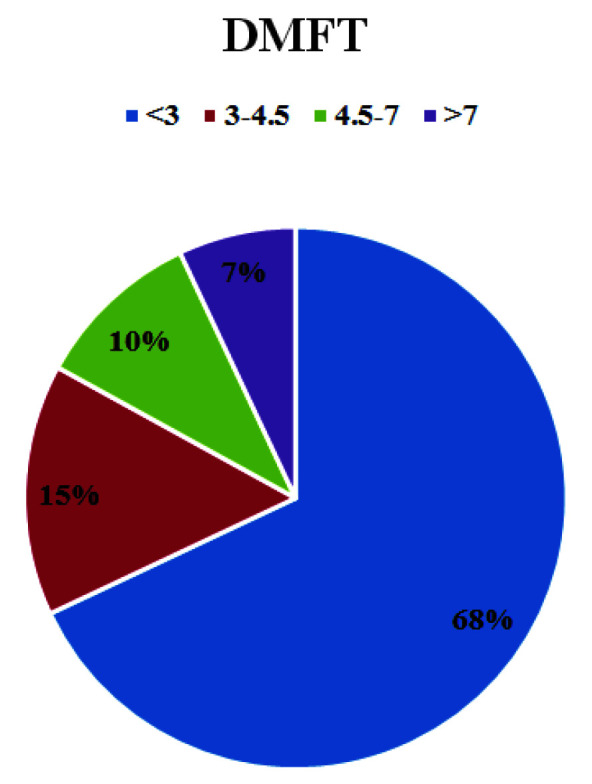
Frequency distribution based on caries activity

The average age of the participants was 13.72 ±0.74 years. Mean and standard deviation of sAA and DMFT were 1326.56±4.73 U/L and 2.77±2.36, respectively. The details were shown in Table 2 and the frequency distribution based on caries activity was shown in Figure 1. Kolmogorov-Smirnov test indicated that neither sAA nor DMFT had the normal distribution (*p*< 0.05). According to Kruskal Wallis test, no significant difference was found among sAA level and DMFT of different BMI percentiles ( Table 3).

**Table 2:** Mean (SD) of the studied variables

Variable	Mean (SD)
Age (year)	13.72 (0.74)
BMI	25.59 (5.05)
DMFT	2.77 (2.36)
D	2.44 (2.12)
M	0.00 (0.00)
F	0.33 (1.09)
sAA (U/L)	1326.56 (4.73)

BMI: Body mass index, DMFT (D, M, F, T): Decayed missing filled teeth; SD: Standard deviation; sAA: Salivary alpha amylase

**Table 3:** Mean (SD) of sAA concentration (U/L) and DMFT of the studied sample divided according to age-sex specific BMI percentiles

BMI percentiles	sAA	DMFT
5-85 (n=27)	(33.25)1330.87	2.55(2.39)
85-95 (n=27)	(53.59)1329.19	3.07(2.30)
95 (n=27) <	(57.03)1319.62	2.70(2.46)
Total (n=81)	1326.56 (4.73)	2.77(2.36)
*p* Value*	0.68	0.71

* According to Kruskal Wallis test, significance level at *p*< 0.05

Abbreviations; BMI: Body mass index, DMFT: Decayed missing filled teeth; SD: Standard deviation; sAA: Salivary alpha amylase

Additionally, the result of Fisher exact test revealed no significant difference in caries activity of different BMI percentiles (*p*= 0.78). Based on Spearman correlation coefficient test, there was a significant positive correlation between DMFT and sAA only in BMI percentile> 95(r: 0.46, *p*= 0.014).

## Discussion

Considering the results of the current study, no significant difference was found between sAA levels in overweight adolescents compared to those who were in “normal” and “at risk for overweight” groups. Although the effect of sAA gene on the risk of obesity has been confirmed, its mechanism remains unclear and there are conflicting results on the association between sAA and obesity [ 19 - 21 ]. Rukh *et al*. [ 23 ] revealed no association between sAA gene copy number and BMI in adults but they indicated that increasing the sAA gene copy number in people with low and high starch intake has a reverse and direct correlation with BMI, respectively. They revealed that obesity in people with high sAA gene copy number can be related to the carbohydrate content in their diet. De Compos *et al*. [ 21 ] assessed the relationship between several salivary components including sAA level in normal, overweight, and obese children aged 5-12 years. They found no significant difference among groups. Although, the studied age range was different, their finding was in accordance with the results of the present study.

Additionally, no considerable difference was found between mean DMFT in the 3 groups of BMIs. This finding is in conformity with the studies of Shakya *et al*. [ 24 ] and Sadeghi *et al*. [ 13 ]. Additionally, the results of the present study confirmed the findings of Heinrich *et al*. [ 25 ], Alves *et al*. [ 26 ], Mojarad and Meybodi [ 27 ], Sadeghi and Alizadeh [ 12 ], and Pinto *et al*. [ 28 ]. Moreover, the results of a systematic review conducted by Silva *et al*. [ 29 ], did not confirm the association between dental caries and obesity. Unlike, Bud *et al*. [ 30 ] and Reddy *et al*. [ 14 ] showed a higher caries incidence in the underweight children aged 6-12 years. They stated that malnutrition in early years of life might influence the enamel development and salivary function leading to increased susceptibility to dental caries. The underweight adolescents were not included in the present study, because based on the preliminary investigation; there were not enough underweight samples in the study area. In contrast, Ghasempour *et al*. [ 15 ] showed that caries activity increased by weight in 3- to 6-year-old children. Furthermore, Sakeenabi *et al*. [ 31 ] found a positive association between caries incidence and overweight in 6- to 12-year-old children. In another study, Willershausen *et al*. [ 32 ] investigated the association of overweight and dental caries in 6- to11-year-old children and concluded that the prevalence of primary and permanent tooth decay was related to overweight. The authors believed that these findings were related to their dietary habit and high amount of carbohydrate intake. Overall, this discrepancy can be explained regarding difference in age range of study population and sample size and characteristics. The present study was conducted on adolescent girls in permanent dentition while the mentioned studies were done on children in primary or mixed dentitions. Whereas dental caries and obesity are multifactorial in nature, contradictory results were predictable and many confounding factors should be taken into account. Moreover, the results should be interpreted carefully because these studies usually are cross sectional; it means BMI might change by time while its effect on teeth could yet remain.

In the present study, a positive correlation was found between sAA level and DMFT only in overweight group. This finding may be related to the level of starch intake by the overweight subjects. As mentioned, excessive consumption of starch by people with higher levels of sAA puts them at risk of obesity [ 23 ]. On the other hand, high carbohydrate intake is related to dental caries [ 13 ] and food habits must be considered for proper interpretation of these findings. Since the current study did not consider the dietary habits, the above findings cannot be interpreted definitively.

The available data concerning the association between dental caries and sAA are limited and inconsistent [ 33 - 36 ]. Mojarad *et al*. [ 33 ] in a matched case-control study assessed the sAA level in 3- to 6-year-old children with and without tooth decay. They found a considerable relationship between low level of sAA and susceptibility to dental caries and declared that although, the study groups were matched for oral hygiene and dietary habits, the other salivary components such as immunoglobulins with possible synergistic or antagonistic effects on sAA were not assessed. Finally, they could not firmly establish a proper sequence between dental caries and sAA. Ahmadi-Motamayel *et al*. [ 34 ] and Sitaru *et al*. [ 35 ] revealed a relationship between sAA and dental caries but Farias *et al*. [ 36 ] did not confirm such a relationship. Ahmadi-Motamayel *et al*. [ 34 ] investigated the relationship between sAA and tooth decay on caries-active and caries-free adolescents with limiting the confounding variables including stress, exercise, smoking, and caffeine intake because of their strong impact on sAA concentration. They concluded that high amount of sAA increases the susceptibility to dental caries by hydrolysis of starch and facilitating the acid production by cariogenic bacteria. Sitaru *et al*. [ 35 ] conducted a research on 10- to 14-year-old children and obtained the same result. They interpreted this finding regarding the impact of sAA on promoting enamel demineralization. Farias *et al*. [ 36 ] carried out a study on 12-47 months caries-active and caries-free children and assessed salivary immunoglobulins A, G, M, total protein, and amylase activity. They found higher levels of salivary immunoglobulins A and G in caries-active children while the amount of salivary immunoglobulin M, total protein and amylase activity were not different between two groups. This divergence may be due to ignoring the other risk factors of dental caries which may have confounding effect on each other [ 33 ].

The current study was carried out with some limitations such as disregarding the dietary habits and stress level of the participants which might have an impact on the level of sAA. In addition, the study was conducted on adolescent girls who were in the pubertal period and puberty may have an impact on body biochemical and lifestyle, which may subsequently influence the study variables. Moreover, because the age of puberty for girls and boys is different, the authors had to study just on girls. Further investigations on all target age groups with a larger sample size in both genders are recommended. Additionally, matching the confounding factors such as oral hygiene, food habits, salivary immunoglobulins, stress level and so on, are suggested for future studies.

## Conclusion

The concentration of sAA and DMFT were not different among overweight and non-overweight adolescent girls. There was a positive correlation between sAA level and DMFT in overweight adolescent girls.
